# Production of AFB1 High-Specificity Monoclonal Antibody by Three-Stage Screening Combined with the De-Homologation of Antibodies and the Development of High-Throughput icELISA

**DOI:** 10.3390/toxins16010011

**Published:** 2023-12-25

**Authors:** Chengchen Pang, Qiang Liu, Lin Chen, Bei Yuan, Chuanyun Zha, Kunying Nie, Haitao Xu, Keyun Ren, Chunlei Yu, Yemin Guo, Qingqing Yang

**Affiliations:** 1 School of Agricultural Engineering and Food Science, Shandong University of Technology, No. 266 Xincun West Road, Zibo 255049, China; 2 Shandong Provincial Engineering Research Center of Vegetable Safety and Quality Traceability, No. 266 Xincun West Road, Zibo 255049, China; 3 Zibo City Key Laboratory of Agricultural Product Safety Traceability, No. 266 Xincun West Road, Zibo 255049, China; 4 Hubei Provincial Institute for Food Supervision and Test, No. 8 Yaojian 2th Road, Wuhan East Lake High-Tech Development Zone, Wuhan 430075, China

**Keywords:** high-specificity AFB1 monoclonal antibody, modified limiting dilution method screening, high-throughput icELISA, immunoassay, increased specificity of cell lines

## Abstract

To achieve accurate detection of AFB1 toxin content in agricultural products and avoid false-positive rates in the assays, the specificity of mAbs is critical. We improved the specificity of the prepared monoclonal antibodies by modifying the traditional limiting dilution subcloning method. The traditional finite dilution method was modified with three-stage screening (the trending concentration of standards used in the screening is low–high–low) to achieve high specificity in pre-cell screening and increased the number of subclones to 10 to achieve the de-homologation of antibodies. A modified limiting dilution obtained a highly specific AFB1 monoclonal cell line, ZFG8, with a 50% inhibition concentration (IC50) of 0.3162 ng/mL. Notably, it exhibited the highest specificity compared to anti-AFB1 monoclonal antibodies prepared by other investigators. The maximum cross-reactivity of the mAb with structural analogues for AFB2, AFG1, AFG2, and AFM1 was 0.34%. The results showed that this type of screening improves the monoclonal antibodies’ specificity. Based on this ZFG8 monoclonal antibody, an icELISA assay was established with an IC50 of 0.2135 ng/mL for AFB1. The limit of the linear detection range of icELISA is 0.0422–1.29267 ng/mL with reasonable specificity and precision. The recoveries of AFB1 in samples of corn flour and wheat meal ranged from 84 to 107%, with CVs below 9.3%. The recoveries of structural analogues (AFB2, AFM1, AFG1, and AFG2) were less than 10% in both corn flour and wheat meal. The results showed that the prepared AFB1 monoclonal antibody could accurately and specifically recognize AFB1 residues in agricultural products while ignoring the effects of other structural analogues.

## 1. Introduction

Aflatoxins (AFs) are secondary metabolites primarily produced by Aspergillus flavus and Aspergillus parasiticus. These mycotoxins are most commonly found in warmer climates and can easily contaminate agricultural products when exposed to hot or humid conditions [1,2]. Furthermore, studies have demonstrated that aflatoxin B1 is an incredibly potent natural carcinogen with a high level of toxicity [3]. Therefore, limited standards or detailed guidelines on AFB1 in food have been developed in different countries and regions worldwide to protect consumer health [4]. AFB1 exhibits remarkable stability in terms of its physicochemical properties, such as high-temperature resistance and solubility in organic solvents [5,6]. To reduce the economic losses and health risks caused by AFB1, simple, rapid, and effective detection methods are a critical part of the process. In recent years, many reported methods have been used for the detection of AFB1 mycotoxins in agricultural products, such as HPLC [7], LC-MS [8], colorimetric immunoassay [9], electrochemistry [10], chemiluminescence [11], electrochemiluminescence methods [12], surface plasmon resonance [13], surface-enhanced Raman scattering [14], immuno-PCR [15,16], etc. At the same time, immunological methods with high specificity for antibody-antigen recognition have been developed and applied in various fields due to their excellent performance [17]. Immunoassays include ELISA, immunochromatography, fluorescence polarization immunoassay, and biosensor assay [18]. ELISA has the advantages of simple operation, high sensitivity, high specificity, and no radioactive contamination. It is suitable for high-throughput field screening and has other advantages [19]. Commercial kits based on them are commonly used in a simple direct or indirect mode and are widely used in food safety.

Monoclonal antibodies are one of the keys to the application of immunoassays. Their quality affects the sensitivity, specificity, and stability of immunoassays. Therefore, the preparation of high-quality monoclonal antibodies remains a bottleneck that needs to be addressed. Most monoclonal antibodies are prepared by hybridoma technology, and the most classical and common screening method is ELISA screening combined with a limiting dilution method for monoclonal positive hybridoma cell lines [20]. For example, Daohong Zhang [21] used a modified ELISA-two-step screening method combined with 2–4 subclones to screen for an ultra-sensitive generic aflatoxin monoclonal antibody 1C11 and two specific monoclonal antibodies against aflatoxin B1 and M1. Yuanyuan Zhang [22] used a hybridoma cell screen with three subclones to obtain 4D9 hybridoma cells that were able to secrete the monoclonal antibody against AFB1 stably. In the conventional method, screening is usually time-consuming and laborious, prone to the loss of positive strains [23].

The traditional limiting dilution subcloning method to screen monoclonal cells can be simply described as the cell plate undergoing a competitive icELISA, and the cells from the positive wells are distributed in a 96-well cell plate so that there is only one cell in each cell well as much as possible to ensure its monoclonal nature. This step is called the subclone of the positive cell wells. The concentration of the standard used in the competition, icELISA, determines the sensitivity and specificity of the cell line obtained. The process of obtaining a monoclonal cell line after multiple finite dilutions is called monoclonal cell screening.

However, in preparing a monoclonal cell line with the traditional limiting dilution subcloning method, monoclonal antibodies against AFB1 small-molecule toxins focus more on their high sensitivity and ignore their high specificity. The monoclonal antibody’s specificity is crucial to accurately detecting AFB1 toxin in agricultural products and avoiding the false positive rate in the detection method. The most classical and common screening method was optimized to improve the quality of obtaining mAbs. There are two main reasons for the low specificity of the antibodies obtained in screening AFB1 toxin. One is that the standards used in traditional screening range from high to low. Higher concentrations of standards have quantitatively more antigenic determinant clusters and cannot target antibodies with high specificity in the pre-screening phase [24]. Secondly, the V-region genes of antibodies are mutated during animal immunization due to the affinity effect of antibodies [25], resulting in hybridoma cell lines after cell fusion producing structurally fractionated antibodies that recognize more antigenic clusters of similar structures of other toxins, leading to a high rate of cross-reactivity with other toxins.

To improve the specificity of the AFB1 monoclonal antibody, we improved the traditional limiting dilution subcloning method for screening monoclonal cells. Firstly, we took the three-stage screening that varied the concentration of standards in a gradient from low to high to low during the screening of monoclonal antibodies. A low concentration of standards was used in the early stages of screening to obtain cell lines that produce highly specific antibodies because low concentrations of standards had fewer antigenic determinants. Antibodies with high specificity bind preferentially to lower concentrations of standards during icELISA screening; therefore, they will be easier to screen out. Then we increased the number of subclones to remove antibodies that identified other toxin structures, achieving the de-homologation of antibodies. Thereby, the specificity of the monoclonal antibody screened was improved. Finally, combinations of commercially and laboratory-available antigenic antibodies were collected, and the effect of different combinations of antigenic antibodies on the potency and sensitivity of the ELISA was investigated. Moreover, to improve the accuracy and sensitivity of the ELISA, an ELISA working standard curve was established under the optimal antigen–antibody combination and optimal working conditions. The sensitivity, specificity, precision, and accuracy of the method were evaluated, and the constructed ELISA was applied to the high-throughput detection of aflatoxin B1 in corn flour and wheat meal and compared with the detection results of HPLC to evaluate its applicability and specificity.

## 2. Results and Discussion

### 2.1. Antiserum Characterization

**Figure 1:**
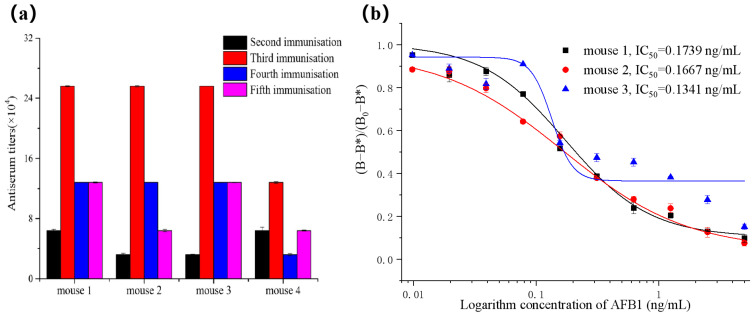
(**a**) Antiserum titers of mice under different immunization times. (**b**) Competition curve of antiserum obtained (*n* = 3).

According to the principle of conventional immunization, a small dose, multiple times, and a long period of immune organisms can make the body continuously produce antibodies against specific antigens. As the number of immunizations increased, it can be seen in Figure 1a that the antiserum titers rose first and then decreased. This may be because B cells are activated in preimmunization in response to antigenic stimulation and cytokines. Memory cells and effector B cells with a sustained immune response are produced, and the titer of IgG increases. When the number of immunizations increases, the body develops immune tolerance to the same immunogen dose, and a titer drop occurs. Generally, titers of 1:16,000 and above were considered acceptable for cell fusion. After five immunizations of mice, the titers of four mice were all higher than 1:64,000, indicating that mice could be used for cell fusion. To obtain a large number of B cells that can specifically recognize AFB1 for cell fusion, producing more specific hybridoma cells. At the corresponding titers, the antisera were tested by competitive ELISA to evaluate the sensitivity of the antibodies.

Moreover, the results in Figure 1b showed that the IC50 values of mice 1, 2, and 3 were not significantly different; the antisera of mouse 4 had no competitive effect. Mouse 3 has the lowest IC50 value, considering mice 1, 2, and 3 (IC20-IC80 values are 0.0582–0.5190, 0.0332–0.8354, and 0.1025–0.1756 ng/mL, respectively). Mouse 2 was chosen for cell fusion due to its wider detection range.

### 2.2. Effect of a Modified Limiting Dilution Method of Subclonal Screening on Antibody Positivity and Specificity

In the antibody-to-antigen recognition process, antibodies recognize antigenic epitopes with a certain structural similarity [24]. Standards at lower concentrations have fewer antigenic determinants and can preferentially bind highly specific antibodies during cell screening. When the binding site on the standard is already sufficient, the less specific antibody will not bind to the standard so that more specific cells can be screened at an earlier stage. Therefore, we took the gradient from low to high to low concentrations of standards and used the high specificity advantage of suitable quality antibodies to screen cells with high specificity in the previous period.

Due to an affinity maturation effect, somatic mutations occurred in the antibody genes produced by the immune system following antigen stimulation [25]. The homology of the antibodies produced after cell fusion (producing antibodies that are structurally similar but with some structural differences) is the main reason for the high cross-reactivity rate of monoclonal antibodies with structural analogues of AFB1. Thus, the monoclonal cell line screening increased the number of subclones to achieve de-homologation of the antibody, making its antibody more precise in recognizing AFB1 toxin than other structural analogues of other AFB1 toxins.

**Figure 2:**
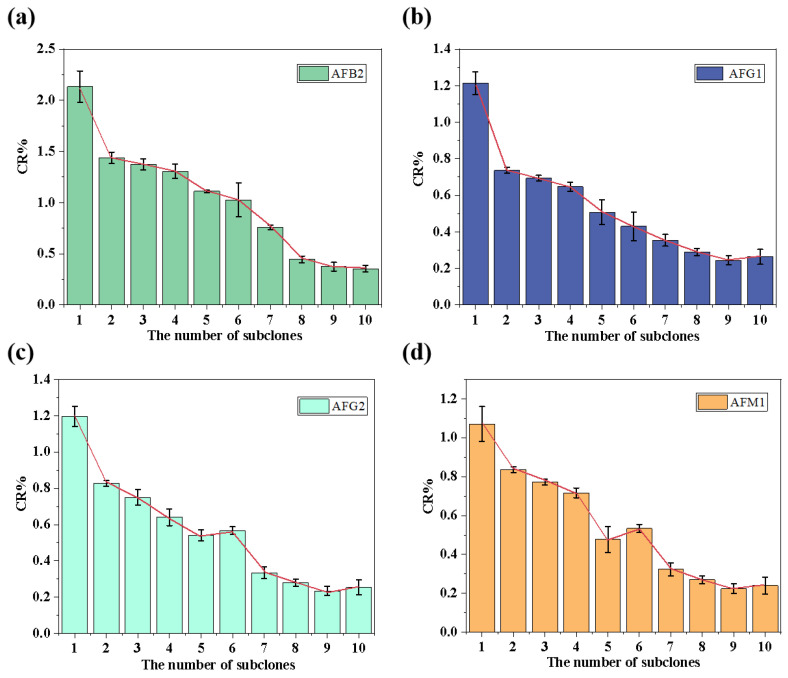
Cross-reactivity of antibodies to structural analogs in the supernatant after each subclone ((**a**–**d**) the cross-reactivity to AFB2, AFG1, AFG2, and AFM1, respectively) (*n* = 3).

**Figure 3:**
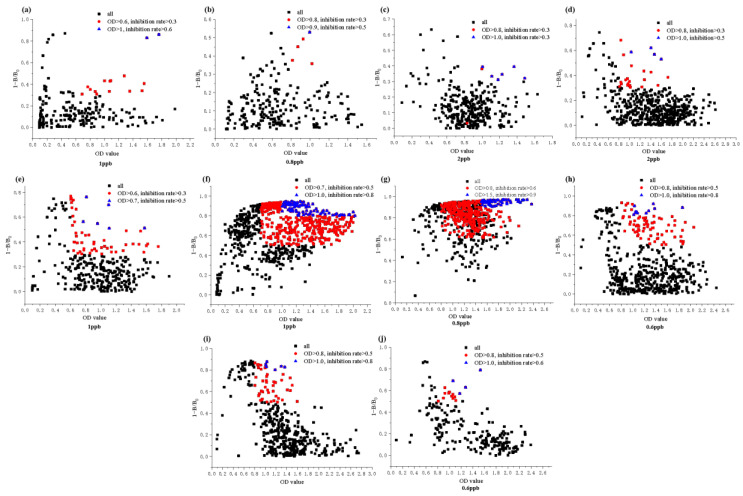
Positive hybridoma cell screened by ELISA ((**a**–**j**) 1st–10th subclone screening, respectively).

As seen in Figure 2 and Figure 3, as the number of screenings increased, the specificity of antibodies in the supernatants of positive hybridoma cells after each subclone increased; the ratio of positive hybridoma cells to the total amount of cells screened increased first and then decreased as the concentration of the standard varied (low–high–low). The standard concentration adopted at the first screening was 1 ppb, and the results indicated a low positive cell ratio and a high specificity of the antibodies in the supernatant. This is because low-concentration standards have fewer antigenic clusters, and more specific antibodies will preferentially bias binding toward standards. We reduced the standard concentration to 0.8 ppb in a second screening, and there was no significant change in the proportion of positive cells, but the specificity of the antibody was improved. In the third to fourth screenings, we raised the concentration of standards so that cells with high specificity began cell enrichment and further culture. The rate of positive cells rose slowly, and there was no significant change in the specificity of the antibody. During the fifth through seventh screenings, the concentration of the standard was slowly reduced to screen out cell lines with high sensitivity and specificity. In the eighth to tenth screening, the specificity of the antibody no longer changed significantly, indicating that its specificity was stable and that hybridoma cell lines with very high specificity had been obtained.

### 2.3. Evaluation of the Specificity of mAbs

**Figure 4:**
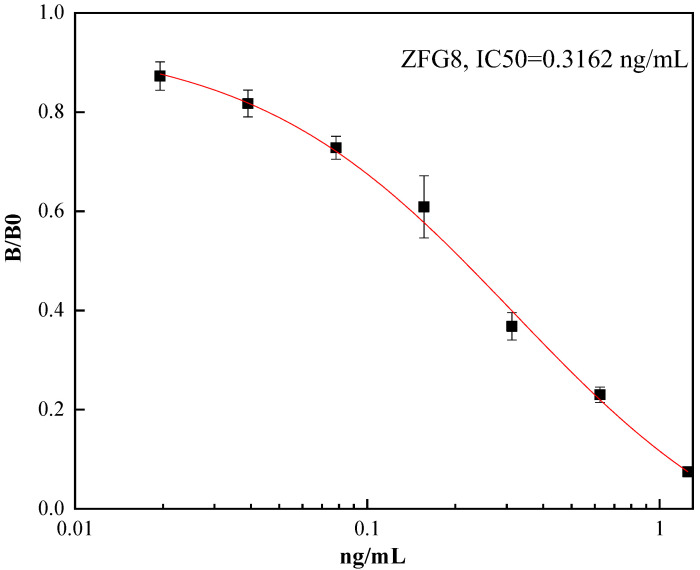
The competition curve of ZFG8 mAb (*n* = 3).

As shown in Figure 4, the IC50 value of mAb was 0.3162 ng/mL, and the detection range was (0.07603–1.31931) ng/mL (IC20–IC80). As shown in Table 1, the IC50 values of the mAbs were 0.3162 ng/mL against AFB1, 182.6005 ng/mL against AFB2, and bigger than 182.6005 ng/mL against other mycotoxins. According to the cross-reactivity formula, the reactivity against AFB2 was 0.34%, and the reactivity against AFM1, AFG1, and AFG2 was lower than 0.34%, demonstrating that the resulting mAbs could recognize AFB1 with high specificity. As shown in Table 2, ZFG8 has the highest specificity compared to AFB1 monoclonal antibodies prepared by other investigators.

### 2.4. Development and Condition Optimization of icELISA for AFB1

**Figure 5:**
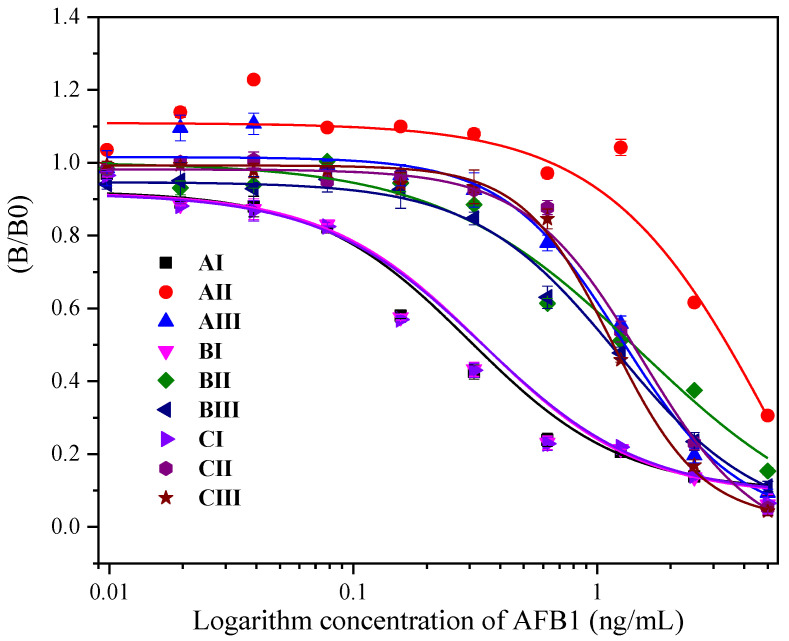
Competition curves under different combinations of antigen and antibody.

In order to select more reasonable antigen–antibody combinations to establish ELISA curves, we collected antibodies and antigens available in the laboratory for cross-reactivity to determine their sensitivity. As shown in Figure 5, it could be concluded from the IC50 values under different antigen–antibody combinations that although the affinity of II (1C11) is high, its sensitivity is the lowest, and its specificity for recognizing AFB1 monoclonal antibodies is weak. As shown in Table 3, the combination of AI and CI had the highest sensitivity. Considering the economic cost, batches of products, reconstituted titers, IC50 values, and the high specificity of the ZFG8 antibody, we chose the combination of CI (antibody: ZFG8, AFB1-BSA: from China Agricultural University Research Institute) to establish ELISA.

**Figure 6:**
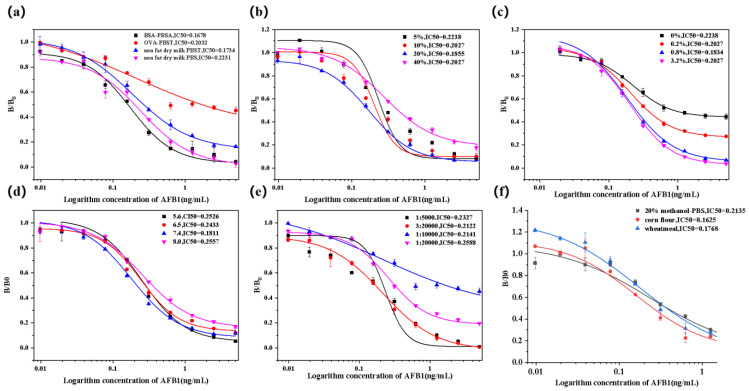
Influence of different working conditions on the sensitivity of icELISA (*n* = 3). (**a**) The competition curves of different kinds of blocking solutions. (**b**) The competition curves of different methanol concentrations. (**c**) The competition curves of different salt ion concentrations. (**d**) The competition curves of different pH conditions. (**e**) The competition curves of the dilutions of different enzyme-conjugated secondary antibodies. (**f**) icELISA curves established in different matrices.

Since antibody-antigen was a reversible equilibrium chemical reaction, the activity of antibodies was influenced by multifaceted factors, which further affected the recognition reaction to antigen. As shown in Figure 6, the effect of different reaction conditions on the sensitivity of icELISA was investigated to obtain the best reaction effect. As shown in Figure 6a, the IC50 was 0.1678 ng/mL under 1% bsa-past blocking and 0.1734 ng/mL under 5% non-fat dry milk PBST blocking, and the blocking effect of the two was not much different. In order to save on economic costs, 5% nonfat dry milk PBST was selected as the optimal blocking solution. As shown in Figure 6b, the IC50 values first decreased and then increased as the methanol concentration increased. The minimum value was reached at 20% methanol-PBS, and the concentration was selected as the optimal reaction condition. As shown in Figure 6c, the sensitivity increased with the increase in salt ion concentration. The sensitivity was highest at 0.8% salt ion concentration (IC50 was 0.1834 ng/mL). Therefore, 0.8% sodium chloride concentration was selected as the optimal salt ion concentration. As shown in Figure 6d, the sensitivity was the highest under the condition of neutral pH 7.4 (IC50 was 0.1811 ng/mL), so 7.4 was selected as the optimal pH condition. As shown in Figure 6e, as the dilution ratio of the enzyme-labeled secondary antibody increased, the sensitivity first increased and then decreased. Due to the slight difference in IC50 values between 3:20,000 and 1:10,000 for enzyme-labeled secondary antibodies, 1:10,000 was selected as the dilution multiple for enzyme-labeled secondary antibodies to save costs.

As shown in Figure 6f, the icELISA curves were established using 20% methanol/PBS, corn flour and wheat meal as substrates under optimal conditions, respectively. Their IC50 values were 0.2135 ng/mL, 0.1625 ng/mL, and 0.1768 ng/mL, respectively; the IC20-IC80 were 0.0422–1.29267 ng/mL, 0.04197–0.62987 ng/mL, and 0.03329–0.93994 ng/mL.

### 2.5. Recovery of the icCLEIA and Correlation between the icCLEIA and UPLC-MS/MS Analysis

To evaluate the specificity and accuracy of the icELISA for AFB1 toxin, spiking experiments with AFB1 toxin and its structural analogues were performed in corn flour and wheat meal to validate the antibody’s highly specific recognition of AFB1. In order to evaluate the specificity and accuracy of AFB1 toxin in icELISA, spiking experiments of AFB1 toxin and its structural analogues were performed in corn flour and wheat meal to validate the high specific recognition of AFB1 by antibodies. AFB1 standard and its structural analogues (AFB2, AFG1, AFG2, and AFM1) with standard concentrations of 0.5, 5, and 10 μg/kg were spiked into the samples identified as negative by UPLC-MS/MS. The samples were extracted and subjected to icELISA and UPLC-MS/MS. As shown in Table 4. In corn flour samples, the mean recovery values of icCLEIA for AFB1 ranged from 84% to 92% with coefficient of variation (CV) values between 1.3% and 9.3%, and UPLC-MS/MS for AFB1 ranged from 94% to 96% with CV values between 1.3% and 6.6%. In wheat meal samples, the mean recovery values of icCLEIA for AFB1 ranged from 93% to 107% with coefficient of variation (CV) values between 0.1% and 1.8%, and UPLC-MS/MS wheat meal for AFB1 ranged from 93% to 98% with CV values between 2.2% and 13.6%.

Because of its high specificity for AFB1 toxin, the recoveries of structural analogues of AFB1 (AFB2, AFG1, AFG2, and AFM1) were less than 10% in both corn flour and wheat meal, which ensured the accurate detection of AFB1 concentration in the samples. The influence of other structural analogues and false positives can be avoided.

## 3. Conclusions

In this experiment, the traditional limiting dilution subcloning method for obtaining monoclonal antibodies was improved. Three-stage screening (the standard concentration was low–high–low) achieved a high specificity of cell lines in the pre-screening phase, and subcloning the positive cell line 10 times achieved the de-homogenization of antibodies. Based on the modified traditional limiting dilution subcloning method, a specific hybridoma cell line, ZFG8, was produced in this study that secreted a highly specific monoclonal antibody to AFB1 with an IC50 of 0.3162 ng/mL and a detection range of 0.07603–1.31931 ng/mL (IC20–IC80). The maximum cross-reactivity of the monoclonal antibody against other aflatoxins (AFB2, AFG1, AFG2, and AFM1) was 0.34%, and its specificity was significantly improved compared to the AFB1 monoclonal antibodies prepared by other investigators, indicating that the screening approach adopted in this study did improve the specificity of the AFB1 monoclonal antibody cell lines prepared. An icELISA working curve was established based on the ZFG8 monoclonal antibody, and its reaction conditions were optimized. The recoveries of AFB1 in samples of corn flour and wheat meal ranged from 84 to 107%, with CVs below 9.3%. Due to the high specificity of the AFB1 monoclonal antibody, the recoveries of AFB1 structural analogs (AFB2, AFG1, AFG2, and AFM1) were less than 10% in both wheat meal and corn flour. The established ELISA showed high agreement with HPLC, indicating that the proposed method had high accuracy and applicability. The above studies provide a certain reference for screening methods and mAb preparation for high-specificity hybridoma cells.

## 4. Materials and Methods

### 4.1. Main Reagents and Consumables

BSA, OVA, Freund’s complete/incomplete adjuvant, HEPES, L-Glu, HT/HAT (50×), PEG1450, and DMSO (bio-sterile grade) were purchased from Sigma, (St. Louis, MO, USA). AFB1, AFB2, AFM1, AFM2, AFG1, and AFG2 were purchased from Tianjin Alta. Double antibodies (penicillin) (100×), non-essential (100×), were purchased from Thermofisher, (Waltham, MA, USA). RPMI-1640 basal medium was purchased from Cytiva, (Beijing, China). Growth factors were purchased from Beijing Biodragon Immunotechnologies Co., Ltd, (Beijing, China). FBS was purchased from Zhejiang Tianhang Biotechnology, (Zhejiang Province, China). Goat anti-mouse IgG-HRP (2 mg/mL) was purchased from Shandong Lvdu Bio-science & technology CO., Ltd (Binzhou, China). AFB1-BSA was purchased from sigma, BeijingBoaolong Immunotech, USA, and provided by the Research Institute of China Agricultural University, respectively. The anti-AFB1 monoclonal antibody 1c11 was provided by the Research Institute of China Agricultural University and purchased from Shandong Lvdu Bio-science & technology Co., Ltd. The mouse myeloma cell line is SP2/0 provided by Shandong Fenghua Biological Co., Ltd, (Zibo, China). All other reagents were analytically pure. The 96-well polystyrene enzyme plates (3590) and cell culture plates (3599) were purchased from Costar, (New York, NY, USA).

### 4.2. Main Instruments

An amalgamator (HL-AH) was used to mix the immune reagents and was purchased from Hangzhou Zhongrun Medical Equipment, (Zhejiang Province, China). The enzyme labeler (Varioskan LUX), plate washer (888), high-speed frozen centrifuge (Sorvall ST 16R), and CO_2_ cell incubator (1N/PE, 5% CO_2_, 37 °C) were purchased from Thermofisher. Pipette guns were purchased from Eppendorfs, (Hamburg Germany). The inverted microscope (BDS400) was purchased from Chongqing Aote Optical Instruments, (Chongqing, China). The analytical balance (AL 104) was purchased from Mettler Toledo Instruments, (Columbus, OH, USA). And the ultrapure water (18.2 MΩ·cm) used was prepared by an ultrapure water instrument was purchased from PALL Corporation (Washington, DC, USA).

### 4.3. Animal Immunity and Cell Fusion

The SPF-grade female Balb/c mice (4–6 weeks) were ordered by Jinan Pengyue Experimental Animal Breeding Co., Ltd. The certificate number is 370726230100840715, and the license number is SCXK (Lu) 20220006. The study respected the lives and rights of animals, followed human social morality, and there was no abuse of laboratory animals or violation of ethics.

After repeated slow aspiration of the emulsified AFB1 antigen with a 1 mL sterile syringe and expulsion of air, the immunization reagent was injected into the back of the neck of mice at 4–5 spots each. The animal immunization strategy is shown in Table 5. After the second immunization, blood was taken from the tails of the mice on day 7 after each immunization to obtain the antisera. Indirect ELISA and indirect competitive ELISA detected antibody titers and the sensitivity of the antisera. Data were processed by OriginPro 9.0. Mice with high titer and low IC50 were selected, and their spleen cells were dissected and fused with mouse myeloma cells sp2/0 (1–2×10^7^ cells) by polyethene glycol (PEG) to prepare hybridoma cells.

### 4.4. Hybridoma Cell Screening and Changes in Antibody Specificity in the Supernatant

The fused cells were gently resuspended into HAT medium, mixed well, and inoculated onto 96-well cell culture plates at approximately 150–200 uL/well. The cells were incubated at 37 °C in a 5% CO_2_ incubator until about day 7. Each well was filled with HT medium at 1 drop/well to promote the growth and multiplication of hybridoma cells. On the 14th day or so, faint spots could be seen on the base of the culture, and the medium changed from purplish red to yellow. The number of cells observed by microscopic examination was 1/4–1/3 of the culture wells, at which point the cell supernatant could be detected by indirect competitive ELISA. Positive wells were selected, and the cells were subcloned by the limiting dilution method. After the first three subclones, the hybridoma cells were cultured in 2%, 1%, and 0.5% HT medium, respectively, followed by RPMI-1640 complete medium. On day 4, after subcloning, cell colonies were visible on microscopic examination. On day 7, after subcloning, the RPMI-1640 complete medium was replenished at 1 drop/well. On or around day 14, the cells grew to about 1/4 of the cell well, at which point the culture was slightly yellowed and the cell supernatant assay could be repeated. The concentration of the standards during the monoclonal cell screening was varied as follows: 1–0.8–2–2–1–1–0.8–0.6–0.6–0.6 (ng/mL). Positive cells were subcloned 10 times.

After each subclone, the specificity of AFB1 monoclonal antibody in the supernatant of positive hybridoma cells was determined by competitive icELISA using the structural analogue standard of AFB1 (AFB2, AFG1, AFG2, AFM1).

### 4.5. Preparation and Evaluation of Monoclonal Antibodies

The positive hybridoma cells of the definitive strain were expanded, cultured, and injected intraperitoneally into female mice. The ascites were collected, and the monoclonal antibody (ZFG8) was prepared by purification using the octanoic acid-saturated ammonium sulfate method. Indirect ELISA evaluated the titer and sensitivity of the monoclonal antibodies. Other species of aflatoxins (AFB2, AFM1, AFG1, and AFG2) were detected by indirect competitive ELISA, and the data were processed by origin pro9.0 to obtain IC50 values and calculate the cross-reactivity rate to evaluate the specificity of the monoclonal antibodies. Cross-reactivity rate (CR%) = (a/b) × 100%. Where a refers to the IC50 value of AFB1 and b refers to the IC50 values of other aflatoxin structural analogues.

### 4.6. Optimization of Optimal Antigen–Antibody Combinations and icELISA Working Conditions

The AFB1-BSA antigen was coated in 96-well enzyme-labeled plates at 100 μL/well, and the buffer solution was CBS solution. After the plate was refrigerated at 4 °C overnight, each well was washed 3 times, and then 200 μL/well of blocking solution was added. After incubation at 37 °C for 2 h, each well was washed 3 times, and 50 μL/well of AFB1 standard and 50 μL/well of AFB1 monoclonal antibody were added successively. It was incubated at 37 °C for 1 h, washed each well 3 times, and then added goat anti-mouse IgG-HRP 100 μL/well. After incubation at 37 °C for 1 h, each well was washed 6 times, and then TMB color development solution was added at 100 μL/well. After 15 min of color development, termination solution was added at 50 μL/well. The final OD450 value was read on the enzyme labeler.

AFB1-BSA (A (from sigma, USA), B (from Beijing Biodragon Immunotechnologies Co., Ltd.), C (from China Agricultural University Research Institute)), and anti-AFB1 monoclonal antibodies (I(ZFG8 from this study), II(1C11), III (Shandong Lvdu Bio-science & Technology Co., Ltd.)) were collected. Antibodies and antigens from different sources were tested with their IC50 values and titers to select the best combination, and the icELISA method was established by this combination. The types of blocking solution, different concentrations of methanol PBS, the salt concentrations, the pH values, and the dilution times of the enzyme-labeled secondary antibody PBST solution were optimized to increase the sensitivity of determination. The corresponding index with the lowest IC50 value was selected as the best reaction condition for processing the data by OriginPro 9.0.

### 4.7. Method Establishment and Evaluation of Sample

Corn flour and wheat meal were commercially purchased. Low, medium, and high concentrations of AFB1 and its structural analogue standards were added to samples identified as negative by the large-scale instrument at final concentrations of 0, 0.5, and 10 μg/kg (below, equal to, and above the EU requirements, respectively). Five grams of sample was weighed in a 50 mL centrifuge tube and mixed with 20.00 mL of 70% methanol in water (*v*/*v*) by vortexing. This was placed under ultrasound for 20 min and centrifuged at 6000 R/min for 10 min. The supernatant was filtered twice using a 0.45 uM organic filter membrane. The supernatant was then diluted 5 times with 0.01 M, pH 7.4 phosphate buffer solution, and used. The spiked recoveries and coefficients of variation were calculated to evaluate the precision and accuracy of the method. The microplate reader determined OD450, and Y = (B − B*)/(B0 − B*) was brought into the linear fitting equation to invert out X. The concentration of AFB1 in the sample, C (ng/mL) = 10X. HPLC assayed the same corn flour and wheat meal, and the results were compared to verify the accuracy and reliability of the icELISA results. (Note: B* refers to the blank value, B0 refers to the non-competitive value, and B refers to the competitive value.)

## Figures and Tables

**Table 1:** Cross-reactivity of the anti-AFB1 mAb with AFB1 analogs.

Competitive Analogues	IC50 (ng/mL)	Cross-Reactivity (%)
AFB1	0.3162	100
AFB2	182.60	0.34
AFM1	<182.60	<0.34
AFG1	<182.60	<0.34
AFG2	<182.60	<0.34

**Table 2:** The sensitivity and cross-reactivity rate of existing anti-AFB1 mAbs and anti-aflatoxin universal antibodies.

Positive Strains	Measure	Monoclonal Antibody Type	IC50 (ng/mL)				CR (%)						Time	Reference
			AFB1	AFB2	AFG1	AFG2	AFM1	AFB1	AFB2	AFG1	AFG2	AFM1		
7A1	icELISA	Specificity	0.04	0.15	0.40	0.45	0.40	100	26.7	10	8.9	10	2016	[26]
4D12		Generality	0.02	0.10	0.10	0.09	0.10	100	20	20	22.2	20		
357	icELISA	Specificity	0.003	0.007	0.0034	0.016	a	100	42	88	19	a	2008	[27]
9c7c11	cdELISA	Specificity	0.045	0.057	2.530	2.120	a	100	78.9	1.78	21.2	a	2016	[28]
34	cdELISA	Specificity	0.62	12.4	2	25.8	a	100	5	31	2.4	a	2006	[29]
1NP-D	cdELISA	Specificity	0.037	0.123	0.063	0.463	0.925	100	30	59	8	4	2016	[30]
2A4	icELISA	Specificity	0.12	1.3	0.8	10.3	3.1	100	9.2	15	1.2	3.9	2016	[31]
5D3	icELISA	Specificity	2	280	300	1050	86	100	0.71	0.67	0.19	2.33	2006	[32]
1C11	icELISA	Generality	0.0012	0.0013	0.0022	0.018	0.013	100	92.3	54.5	6.7	9.0	2009	[21]
4F12		Specificity	0.086	0.095	0.101	0.416	0.201	100	90	84	20.7	42.8		
ZFG8	icELISA	Specificity	0.34	182.6	>182.6	>182	>182	100	0.34	<0.34	<0.34	<0.34	2022	b

‘a’ indicates that the IC50 of the toxin was not tested, ‘b‘ Indicates the monoclonal antibody prepared by this study.

**Table 3:** Titers and IC50 under different combinations of antigen and antibody.

	A	B	C
I	1:1000 ^a^, 0.3256 ^b^	1:1000, 0.3383	1:1000, 0.3194
II	1:2000, 5.6755	1:4000, 1.5024	1:4000, 1.5173
III	1:1000, 1.2563	1:1000, 1.2081	1:1000, 1.1535

^a^ refers to the titer under the antigen antibody combination; ^b^ refers to the IC50 value under the antigen antibody combination. The columns show different AFB1 antigens (A (from sigma, USA), B (from Beijing Biodragon Immunotechnologies Co., Ltd.), and C (from China Agricultural University Research Institute)). The rows show different anti-AFB1 monoclonal antibodies (I (ZFG8), II (1C11), III (Shandong Lvdu Bio-science & Technology Co., Ltd.)).

**Table 4:** Recovery of AFB1 and the structural analogues from samples determined by icCLEIA and UPLC-MS/MS (*n* = 3).

Sample: Corn Flour	Spiked (μg/kg)	ELISA Measured ± SD	Recovery (%)	CV (%)	UPLC-MS/MS Measured ± SD	Recovery (%)	CV (%)
AFB1	0.5	0.451 ± 0.042	90	9.3	0.475 ± 0.031	94	6.6
	5	4.636 ± 0.271	92	5.8	4.893 ± 0.124	97	2.5
	10	8.453 ± 0.114	84	1.3	9.626 ± 0.126	96	1.3
AFB2	0.5	0.146 ± 0.022	29	15.1	0.488 ± 0.039	96	8.1
	5	1.551 ± 0.063	31	4.1	5.039 ± 0.127	100	2.6
	10	2.458 ± 0.071	24	2.8	9.514 ± 0.115	95	1.2
AFG1	0.5	0.012 ± 0.002	2	16.6	0.413 ± 0.035	82	8.5
	5	0.112 ± 0.006	2	5.3	4.911 ± 0.141	98	2.8
	10	0.265 ± 0.012	2	4.5	9.217 ± 0.144	92	1.5
AFG2	0.5	0.011 ± 0.001	2	9.0	0.526 ± 0.019	104	3.9
	5	0.133 ± 0.007	2	5.2	4.919 ± 0.232	98	4.7
	10	0.228 ± 0.017	2	7.4	9.655 ± 0.174	96	1.8
AFM1	0.5	0.014 ± 0.002	2	14.2	0.453 ± 0.017	90	3.7
	5	0.116 ± 0.013	2	11.2	4.832 ± 0.066	96	1.3
	10	0.172 ± 0.004	1	2.3	9.215 ± 0.184	92	2.0
**Sample:** **Wheat meal**	**Spiked (μg/kg)**	**ELISA** **measured ± SD**	**Recovery (%)**	**CV (%)**	**UPLC-MS/MS** **measured ± SD**	**Recovery (%)**	**CV (%)**
AFB1	0.5	0.535 ± 0.012	107	1.8	0.498 ± 0.023	98	2.3
	5	4.782 ± 0.026	95	0.4	4.684 ± 0.021	93	2.2
	10	9.327 ± 0.011	93	0.1	9.583 ± 0.131	95	13.6
AFB2	0.5	0.043 ± 0.005	8	11.6	0.562 ± 0.091	100	9.1
	5	0.564 ± 0.004	11	0.7	5.674 ± 0.087	113	7.6
	10	1.388 ± 0.008	13	0.5	9.598 ± 0.012	95	1.2
AFG1	0.5	0.017 ± 0.002	3	11.7	0.483 ± 0.031	96	3.2
	5	0.092 ± 0.012	1	13.0	4.691 ± 0.024	93	2.5
	10	0.288 ± 0.004	2	1.3	9.567 ± 0.141	95	14.7
AFG2	0.5	0.013 ± 0.001	2	7.6	0.515 ± 0.016	102	3.1
	5	0.163 ± 0.013	3	7.9	4.669 ± 0.011	93	1.1
	10	0.193 ± 0.011	1	5.7	9.632 ± 0.021	96	2.1
AFM1	0.5	0.018 ± 0.002	3	11.1	0.529 ± 0.012	104	2.3
	5	0.212 ± 0.013	4	6.1	4.703 ± 0.011	94	1.1
	10	0.537 ± 0.012	5	2.2	9.572 ± 0.123	95	1.2

**Table 5:** Immune strategies of mice at different stages.

Immunization Sequence	Interval (Day)	Immune Dose (μg/one)	Part/Method	Type of Adjuvant
I	0	40	Dorsal multisite injection	FCA
II	28	40	Same as above	FIA
III	49	40	Same as above	FIA
IV	70	40	Same as above	FIA
V	91	40	Same as above	FIA
Shock immunization	3 days before cell fusion	30	intraperitoneal injection	No adjuvant

## Data Availability

Data are contained within the article.
